# Association of Search Query Interest in Gastrointestinal Symptoms With COVID-19 Diagnosis in the United States: Infodemiology Study

**DOI:** 10.2196/19354

**Published:** 2020-07-17

**Authors:** Anjana Rajan, Ravi Sharaf, Robert S Brown, Reem Z Sharaiha, Benjamin Lebwohl, SriHari Mahadev

**Affiliations:** 1 Department of Gastroenterology and Hepatology Weill Cornell Medicine New York, NY United States; 2 Division of Digestive and Liver Disease Columbia University Medical Center New York, NY United States

**Keywords:** COVID-19, diarrhea, internet search queries, Google Trends, gastrointestinal, symptom, health information, pandemic, infectious disease, virus

## Abstract

**Background:**

Coronavirus disease (COVID-19) is a novel viral illness that has rapidly spread worldwide. While the disease primarily presents as a respiratory illness, gastrointestinal symptoms such as diarrhea have been reported in up to one-third of confirmed cases, and patients may have mild symptoms that do not prompt them to seek medical attention. Internet-based infodemiology offers an approach to studying symptoms at a population level, even in individuals who do not seek medical care.

**Objective:**

This study aimed to determine if a correlation exists between internet searches for gastrointestinal symptoms and the confirmed case count of COVID-19 in the United States.

**Methods:**

The search terms chosen for analysis in this study included common gastrointestinal symptoms such as *diarrhea*, *nausea*, *vomiting*, and *abdominal pain*. Furthermore, the search terms *fever* and *cough* were used as positive controls, and *constipation* was used as a negative control. Daily query shares for the selected symptoms were obtained from Google Trends between October 1, 2019 and June 15, 2020 for all US states. These shares were divided into two time periods: pre–COVID-19 (prior to March 1) and post–COVID-19 (March 1-June 15). Confirmed COVID-19 case numbers were obtained from the Johns Hopkins University Center for Systems Science and Engineering data repository. Moving averages of the daily query shares (normalized to baseline pre–COVID-19) were then analyzed against the confirmed disease case count and daily new cases to establish a temporal relationship.

**Results:**

The relative search query shares of many symptoms, including *nausea*, *vomiting*, *abdominal pain*, and *constipation*, remained near or below baseline throughout the time period studied; however, there were notable increases in searches for the positive control symptoms of *fever* and *cough* as well as for *diarrhea*. These increases in daily search queries for *fever*, *cough*, and *diarrhea* preceded the rapid rise in number of cases by approximately 10 to 14 days. The search volumes for these terms began declining after mid-March despite the continued rises in cumulative cases and daily new case counts.

**Conclusions:**

Google searches for symptoms may precede the actual rises in cases and hospitalizations during pandemics. During the current COVID-19 pandemic, this study demonstrates that internet search queries for *fever*, *cough*, and *diarrhea* increased prior to the increased confirmed case count by available testing during the early weeks of the pandemic in the United States. While the search volumes eventually decreased significantly as the number of cases continued to rise, internet query search data may still be a useful tool at a population level to identify areas of active disease transmission at the cusp of new outbreaks.

## Introduction

The coronavirus disease (COVID-19) pandemic has resulted in over 10.3 million cases and over 508,000 deaths to date worldwide [1]. Almost all known information regarding symptoms of COVID-19 has been obtained from studies of patients who seek medical care; fever, cough, fatigue, and dyspnea are the predominant symptoms [2,3]. Early reports have suggested that gastrointestinal symptoms are also a primary manifestation in 3% to 37% of patients, and these symptoms may precede clinical diagnosis [2,4,5]. To study the presentation of COVID-19, clinicians have primarily used the traditional approach of identifying symptom prevalence among confirmed cases [6]. However, due to limited testing availability and the high occurrence of subclinical and minimally symptomatic disease, innovative uses of internet-based approaches may have increased utility in examining symptom manifestations in the general population.

Infodemiology is an emerging field that involves analyzing information from internet sources to obtain insight into changes in population health that may ultimately inform public health and policy, especially during outbreaks and epidemics [7]. Examples of such metrics include dissecting content from Twitter to understand attitudes and behaviors during the Zika virus and Ebola virus outbreaks and exploring the role of media awareness of Middle Eastern respiratory syndrome coronavirus (MERS-CoV) and case management [8-10]. One validated approach includes analyzing internet search queries that reflect the health information–seeking activity of users. This methodology has correlated antecedent symptoms with norovirus outbreaks and has accurately predicted symptom-based patterns of influenza spread and incidence [11-13]. The aim of this infodemiology study was to examine trends of internet search queries for gastrointestinal symptoms during a period of COVID-19 case confirmation within the US population.

## Methods

### Data Sources

Google Trends provides access to an unbiased sample of Google searches. The Google Trends interface reports a “query share,” calculated by dividing the number of queries of interest by the total number of queries for all search terms over the same time period and region. Each query share is normalized on a scale of 0 to 100, with 100 representing the maximum value of the share for the period and region selected [14]. The scaled query share values are plotted daily, generating a time series.

The chosen search terms were gastrointestinal symptoms that have previously been reported to be associated with COVID-19 infection in the literature, including *diarrhea*, *nausea*, *vomiting*, and *abdominal pain*. The terms *fever* and *cough* were included as positive controls. The term *constipation* was included as a negative control, as we felt this symptom was unlikely to be associated with COVID-19. The terms *anosmia*, *dysgeusia*, *loss of appetite*, *loss of taste*, and *loss of smell* were considered; however, due to the low frequency of searches for these terms, analysis was limited by missing data. The default “All categories” and “Web search” settings were selected for the Google Trends query.

Daily case counts of confirmed COVID-19 cases for each US state were obtained from the Johns Hopkins University Center for Systems Science and Engineering data repository [15].

### Data Analysis

Daily query shares for the selected symptoms were obtained from October 1, 2019 to June 15, 2020 for the United States. The full data set of search query shares is provided in Multimedia Appendix 1. The data were divided into two time periods for comparison: a baseline period during which the COVID-19 case burden was low (October 1 to February 29) and a post–COVID-19 period (March 1 to June 15). The query share for each symptom was divided by its average for the pre–COVID-19 period to generate a curve of search interest relative to the pre–COVID-19 baseline. To examine longer-term patterns, the search query shares for the 5-year period preceding the COVID-19 pandemic were plotted. A 3-day moving average smoother was applied to reduce day-to-day variation. Cumulative and new COVID-19 cases from the United States were superimposed on Google search data to assess their temporal relationship with the symptoms. All analyses were performed with Stata 13.0 (StataCorp LP).

## Results

**Figure 1:**
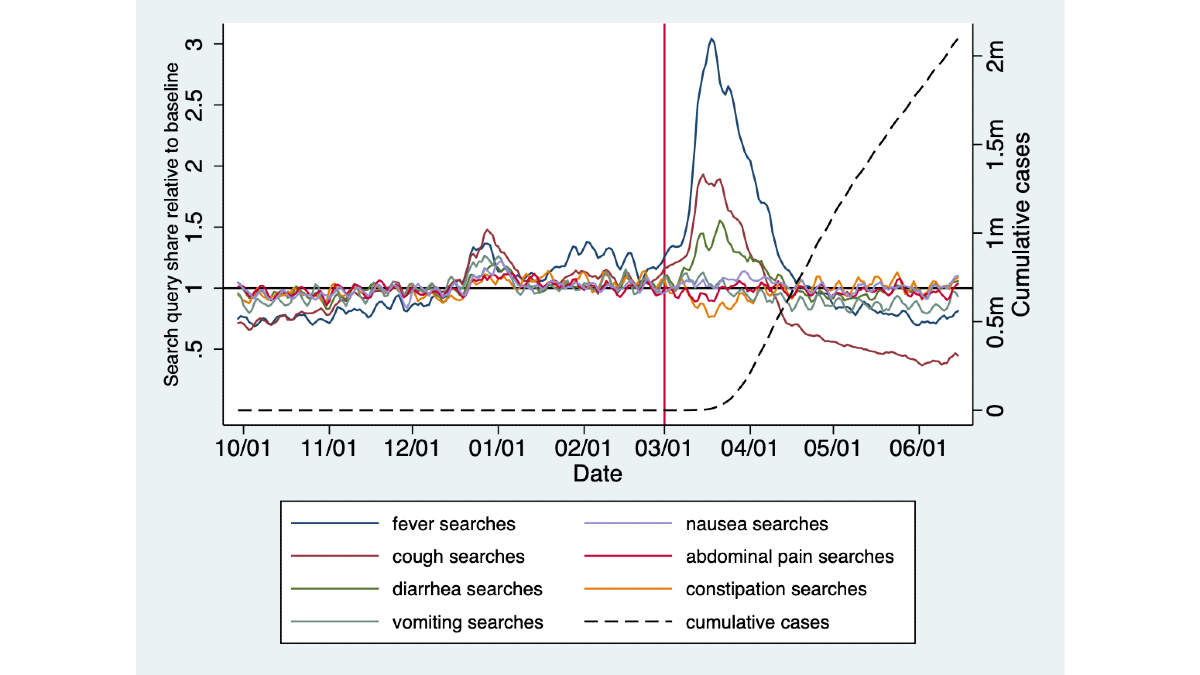
Google search query shares for gastrointestinal symptoms, fever, and cough relative to the pre-March 1, 2020 baseline and their relationships to the cumulative confirmed COVID-19 case count in the United States from October 2019 through June 2020. m: million.

2.1 million cases of COVID-19 were reported within the United States through June 15, 2020. Figure 1 demonstrates a sharp increase relative to the pre–COVID-19 baseline in search query shares for *fever* and *cough* starting on March 7. This trend precedes the rise in reporting of confirmed COVID-19 cases that occurs 10 to 14 days afterward. Notably, the *diarrhea* search query share also increases at the same time or slightly after those for *fever* and *cough*. The search query shares for the remaining gastrointestinal symptoms are either only very slightly above baseline (*nausea* and *vomiting*) or below baseline (*abdominal pain* and *constipation*). The search query shares for *fever*, *cough*, and *diarrhea* all appear to decline after March 20 despite a continued steady rise in cumulative cases through June 15.

**Figure 2:**
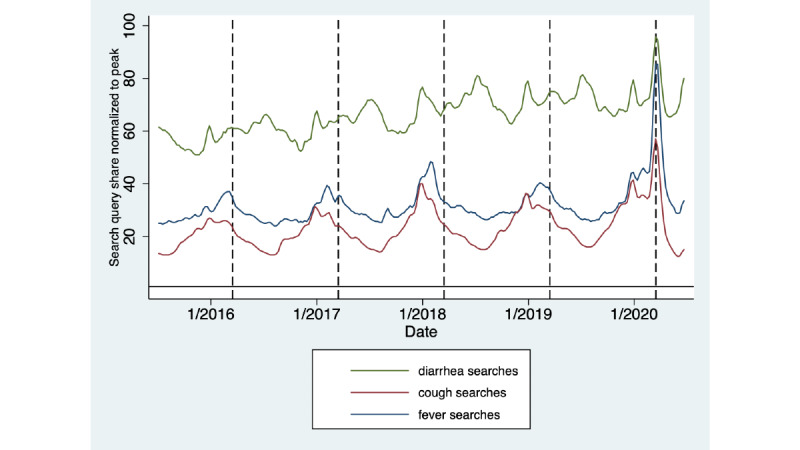
Seasonal trends in Google search query shares for *fever*, *cough*, and *diarrhea* over the last five years as percentages of peak interest.

Figure 2 depicts long-term trends in the query shares for the *fever*, *cough*, and *diarrhea* search terms over a 5-year period. Winter seasonality in the search query shares for all terms is apparent; however, the mid-March peak seen in 2020 in the setting of the COVID-19 pandemic deviates from the decreasing trend at the same point in prior years. As shown in Multimedia Appendix 2, when the new case rate began to trend downward in the first week of April, relative query shares for *fever*, *cough*, and *diarrhea* were already declining, and they returned to or decreased below baseline by mid-April.

## Discussion

### Principal Findings

Our analysis of aggregate internet search query data reveals that the search query shares for symptoms associated with COVID-19 rose in advance of the substantial increase in identified cases that occurred with the first wave of the pandemic in the United States in early March 2020. The data suggest that symptoms of fever, cough, and diarrhea may occur contemporaneously and precede case identifications by up to two weeks in the United States, particularly during the early weeks of the pandemic. This study validates the findings of Higgins et al [16] that COVID-19–related internet searches preceded case identification by over a week in China, Italy, Spain, Washington, and New York.

This study also suggests that there was no significant increase in abdominal pain or constipation queries, which may provide reassurance to clinicians who are faced with these very common complaints in the setting of a new and uncertain pandemic.

The seasonal increase in search query shares for *fever*, *cough*, and *diarrhea* in December 2019 and at the same time in prior years can be attributed to increased search interest during the typical cold and influenza season in the winter. These query shares are much lower than those seen during the post–COVID-19 time range in this study.

Despite the consistent increase in cumulative case count throughout April and May, our findings show that search queries for *fever*, *cough*, and *diarrhea* begin decreasing in mid-March, when the new daily case rate was over 5000 and continuing to rise. There are several possible explanations for the decoupling of COVID-19 cases and search query interest. One explanation is that users sought information via the internet early in the pandemic when there was less public knowledge regarding the virus and its manifestations and that by April, the demand for further information was saturated. During the early weeks of the pandemic, access to outpatient medical care and COVID-19 testing were limited; however, later in the pandemic, both testing and access to telehealth visits became more common, and individuals may thus have relied on alternative sources of information. Our study suggests that internet search query data can provide early clues to the start of an outbreak but may have less utility as the course of the pandemic extends.

### Limitations

There are many limitations and assumptions that must temper our interpretation of these data. Through this infodemiological approach, data were only gathered from internet users, who may not reflect the entire population, such as younger or older persons. Moreover, individuals may be searching for these terms for reasons other than being symptomatic themselves. The role of media attention in influencing user behavior should also be considered. However, public knowledge of the gastrointestinal symptoms associated with COVID-19 was minimal during the period in which the search volumes rose and peaked, which suggests that search interest in diarrhea was less likely to be influenced by media reporting of diarrhea as a manifestation of the disease.

### Conclusions

This study demonstrates sharp increases in internet search interest in fever, cough, and diarrhea at the onset of the COVID-19 pandemic in the United States preceding case identification. Further work is warranted to determine if infodemiological approaches can contribute to population-based surveillance of early outbreaks.

## Appendix

Multimedia Appendix 1 Data set of daily search query shares for the selected symptoms and cumulative and daily case counts.

Multimedia Appendix 2 Google search query shares for gastrointestinal symptoms, fever, and cough relative to the pre-March 1 baseline and their relationships with the daily new case count in the United States.

## Abbreviations

COVID-19: coronavirus disease
MERS-CoV: Middle Eastern respiratory syndrome coronavirus
