# Supplementary material for: Association of Search Query Interest in Gastrointestinal Symptoms With COVID-19 Diagnosis in the United States: Infodemiology Study
Source: JMIR Public Health Surveill. 2020 Jul 17;6(3):e19354. doi: 10.2196/19354 (PMC7371406; doi:10.2196/19354)
Supplement: Multimedia Appendix 1 [file publichealth_v6i3e19354_app1.pdf]

paperanalysis

date	fever	cough	diarrhea	vomiting	abdopain	constipation	nausea	cumcases	postmarch	newcases
01oct2019	28	22	58	17	14	29	31		0	0
02oct2019	29	22	56	17	14	30	29		0	0
03oct2019	27	21	56	16	13	28	33		0	0
04oct2019	26	20	54	14	13	29	31		0	0
05oct2019	26	23	57	16	12	31	31		0	0
06oct2019	26	23	59	17	12	31	32		0	0
07oct2019	29	23	57	16	15	28	34		0	0
08oct2019	29	22	58	17	15	30	31		0	0
09oct2019	28	22	58	16	14	30	32		0	0
10oct2019	26	23	52	15	13	28	32		0	0
11oct2019	26	22	54	14	13	27	28		0	0
12oct2019	28	22	61	18	13	31	30		0	0
13oct2019	28	25	60	18	12	33	32		0	0
14oct2019	29	23	56	17	14	31	34		0	0
15oct2019	30	25	60	16	15	29	34		0	0
16oct2019	31	23	54	17	14	29	32		0	0
17oct2019	29	24	55	17	14	30	30		0	0
18oct2019	28	24	54	16	14	29	27		0	0
19oct2019	26	23	62	18	12	31	33		0	0
20oct2019	28	24	63	20	14	32	33		0	0
21oct2019	30	27	56	19	12	31	34		0	0
22oct2019	30	26	55	16	14	30	34		0	0
23oct2019	29	24	55	14	15	29	30		0	0
24oct2019	28	23	56	18	12	32	29		0	0
25oct2019	27	23	53	17	12	30	30		0	0
26oct2019	27	25	55	18	13	30	32		0	0
27oct2019	28	25	60	18	12	34	33		0	0
28oct2019	28	25	59	19	14	30	32		0	0
29oct2019	28	27	56	19	15	29	34		0	0
30oct2019	27	26	52	15	15	28	35		0	0
31oct2019	26	23	50	16	12	28	31		0	0
01nov2019	25	23	52	15	11	26	31		0	0
02nov2019	28	27	52	19	12	33	31		0	0
03nov2019	28	28	60	18	11	32	31		0	0
04nov2019	32	28	58	21	15	30	33		0	0
05nov2019	31	28	57	18	14	28	34		0	0
06nov2019	32	29	54	19	13	29	33		0	0

**07nov2019**	31	29	54	18	15	29	29		0	0
**08nov2019**	28	28	53	16	13	31	29		0	0
**09nov2019**	31	30	60	20	13	31	32		0	0
**10nov2019**	31	31	61	20	13	32	34		0	0
**11nov2019**	30	31	59	19	16	29	34		0	0
**12nov2019**	30	29	59	18	15	30	32		0	0
**13nov2019**	32	30	66	19	14	29	29		0	0
**14nov2019**	30	29	59	17	14	32	31		0	0
**15nov2019**	29	29	58	17	13	27	29		0	0
**16nov2019**	30	32	61	20	12	31	30		0	0
**17nov2019**	31	30	63	22	12	33	31		0	0
**18nov2019**	33	32	59	19	14	32	35		0	0
**19nov2019**	34	31	58	20	13	29	36		0	0
**20nov2019**	32	31	59	19	14	29	33		0	0
**21nov2019**	33	31	58	19	14	30	32		0	0
**22nov2019**	31	29	57	17	12	29	33		0	0
**23nov2019**	31	31	58	21	12	32	34		0	0
**24nov2019**	33	33	61	19	11	35	34		0	0
**25nov2019**	33	34	60	20	13	35	36		0	0
**26nov2019**	33	32	63	20	14	30	35		0	0
**27nov2019**	31	32	57	19	16	30	35		0	0
**28nov2019**	27	28	54	18	12	25	28		0	0
**29nov2019**	27	29	51	20	11	29	28		0	0
**30nov2019**	30	33	56	20	11	34	33		0	0
**01dec2019**	31	31	65	19	11	32	31		0	0
**02dec2019**	30	29	60	21	16	25	33		0	0
**03dec2019**	32	32	62	18	15	26	34		0	0
**04dec2019**	34	31	59	20	14	32	34		0	0
**05dec2019**	32	32	53	18	12	30	33		0	0
**06dec2019**	32	29	57	17	14	30	31		0	0
**07dec2019**	31	31	63	19	10	31	33		0	0
**08dec2019**	32	33	60	19	13	32	30		0	0
**09dec2019**	34	31	56	18	12	29	38		0	0
**10dec2019**	34	33	58	19	13	30	34		0	0
**11dec2019**	34	31	56	19	12	31	33		0	0
**12dec2019**	33	31	53	18	12	29	33		0	0
**13dec2019**	32	31	51	17	12	27	31		0	0
**14dec2019**	33	33	56	20	12	28	34		0	0
**15dec2019**	37	33	62	21	13	29	34		0	0

**16dec2019**	39	33	56	19	13	27	33		0	0
**17dec2019**	39	33	59	20	12	30	33		0	0
**18dec2019**	35	34	55	20	11	28	33		0	0
**19dec2019**	36	33	53	17	12	26	30		0	0
**20dec2019**	36	33	56	22	10	28	33		0	0
**21dec2019**	40	38	63	21	10	33	33		0	0
**22dec2019**	43	39	66	23	11	33	36		0	0
**23dec2019**	44	39	69	24	13	33	37		0	0
**24dec2019**	44	44	64	24	13	32	34		0	0
**25dec2019**	42	38	65	22	11	29	34		0	0
**26dec2019**	48	45	70	26	13	34	41		0	0
**27dec2019**	45	44	70	26	13	33	42		0	0
**28dec2019**	47	46	73	25	13	35	40		0	0
**29dec2019**	48	44	69	23	13	35	40		0	0
**30dec2019**	44	43	65	25	16	31	41		0	0
**31dec2019**	43	44	72	23	13	29	40		0	0
**01jan2020**	39	37	67	23	11	30	45		0	0
**02jan2020**	40	42	67	24	14	31	41		0	0
**03jan2020**	39	41	65	22	15	35	38		0	0
**04jan2020**	44	41	71	22	14	34	40		0	0
**05jan2020**	40	39	71	23	14	34	38		0	0
**06jan2020**	38	35	63	19	13	31	37		0	0
**07jan2020**	39	36	64	18	15	34	38		0	0
**08jan2020**	38	35	60	18	13	34	35		0	0
**09jan2020**	38	34	61	18	13	33	36		0	0
**10jan2020**	38	32	61	19	12	33	34		0	0
**11jan2020**	38	34	59	17	13	37	34		0	0
**12jan2020**	41	34	64	19	13	37	33		0	0
**13jan2020**	41	32	61	21	16	32	34		0	0
**14jan2020**	40	33	59	21	14	33	35		0	0
**15jan2020**	41	31	58	21	14	34	36		0	0
**16jan2020**	38	29	57	19	12	34	34		0	0
**17jan2020**	37	31	56	17	13	32	33		0	0
**18jan2020**	39	34	62	19	13	36	34		0	0
**19jan2020**	40	31	63	20	12	35	33		0	0
**20jan2020**	38	33	64	20	12	38	35		0	0
**21jan2020**	41	34	60	23	15	35	36		0	0
**22jan2020**	43	32	61	19	15	32	35	1	0	0
**23jan2020**	42	32	57	19	15	32	34	1	0	0

**24jan2020**	39	31	56	20	12	35	33	2	0	1
**25jan2020**	41	37	64	21	13	38	35	2	0	0
**26jan2020**	42	34	62	21	11	36	31	5	0	3
**27jan2020**	42	33	63	20	14	33	34	5	0	0
**28jan2020**	47	34	60	20	14	33	35	5	0	0
**29jan2020**	44	34	64	20	13	35	34	5	0	0
**30jan2020**	43	34	60	19	13	33	35	5	0	0
**31jan2020**	41	33	60	18	11	32	32	7	0	2
**01feb2020**	45	38	62	23	12	36	31	8	0	1
**02feb2020**	45	33	63	23	12	34	35	8	0	0
**03feb2020**	46	31	57	23	15	30	34	11	0	3
**04feb2020**	48	35	62	21	14	32	37	11	0	0
**05feb2020**	43	36	59	19	14	30	33	11	0	0
**06feb2020**	43	32	65	19	13	30	31	11	0	0
**07feb2020**	43	31	57	19	14	31	34	11	0	0
**08feb2020**	44	36	59	20	13	34	36	11	0	0
**09feb2020**	44	36	65	22	12	36	35	11	0	0
**10feb2020**	45	34	60	23	14	31	37	11	0	0
**11feb2020**	45	36	61	20	14	33	35	12	0	1
**12feb2020**	44	34	62	21	13	33	35	12	0	0
**13feb2020**	42	33	60	20	14	35	33	13	0	1
**14feb2020**	37	29	55	18	11	28	33	13	0	0
**15feb2020**	41	35	63	18	13	33	32	13	0	0
**16feb2020**	44	38	62	21	13	37	39	13	0	0
**17feb2020**	43	34	65	23	15	37	40	13	0	0
**18feb2020**	43	31	59	20	13	34	33	13	0	0
**19feb2020**	43	33	63	19	15	33	33	13	0	0
**20feb2020**	39	33	57	18	13	31	34	13	0	0
**21feb2020**	38	31	56	18	12	32	34	15	0	2
**22feb2020**	38	33	63	19	12	37	33	15	0	0
**23feb2020**	40	34	64	23	13	38	33	15	0	0
**24feb2020**	42	31	63	23	14	33	36	15	0	0
**25feb2020**	42	32	64	20	15	33	36	15	0	0
**26feb2020**	42	33	59	19	14	33	32	15	0	0
**27feb2020**	41	34	57	21	13	30	35	16	0	1
**28feb2020**	41	32	58	17	12	31	33	16	0	0
**29feb2020**	42	34	68	21	11	33	32	24	0	8
**01mar2020**	44	35	67	21	12	36	32	30	1	6
**02mar2020**	47	36	63	21	14	31	36	53	1	23

**03mar2020**	47	35	62	19	13	29	34	73	1	20
**04mar2020**	48	34	63	20	15	31	33	104	1	31
**05mar2020**	44	38	59	17	13	30	33	174	1	70
**06mar2020**	46	37	62	20	14	31	33	222	1	48
**07mar2020**	45	38	67	21	11	34	38	337	1	115
**08mar2020**	49	40	73	23	12	33	38	451	1	114
**09mar2020**	52	39	69	22	13	29	38	519	1	68
**10mar2020**	56	40	69	21	14	30	34	711	1	192
**11mar2020**	60	46	71	20	13	28	36	1109	1	398
**12mar2020**	77	57	74	22	11	25	35	1561	1	452
**13mar2020**	82	59	85	21	12	26	32	2157	1	596
**14mar2020**	90	68	89	23	11	30	39	2870	1	713
**15mar2020**	91	63	84	23	12	29	37	2968	1	98
**16mar2020**	98	61	80	21	10	24	36	4309	1	1341
**17mar2020**	100	62	78	21	11	22	32	6041	1	1732
**18mar2020**	100	60	80	19	11	23	36	8803	1	2762
**19mar2020**	100	62	84	21	11	23	35	14024	1	5221
**20mar2020**	92	61	92	19	13	24	33	19305	1	5281
**21mar2020**	88	62	98	21	13	29	35	25606	1	6301
**22mar2020**	87	60	100	21	12	27	37	33519	1	7913
**23mar2020**	86	55	84	21	12	26	38	43581	1	10062
**24mar2020**	85	54	87	21	12	26	38	53802	1	10221
**25mar2020**	87	52	83	21	13	26	39	65675	1	11873
**26mar2020**	81	50	81	21	14	27	37	83646	1	17971
**27mar2020**	78	49	76	21	13	29	36	101757	1	18111
**28mar2020**	78	51	83	17	13	31	39	121421	1	19664
**29mar2020**	74	49	82	21	12	33	41	140494	1	19073
**30mar2020**	69	43	71	18	11	30	36	161875	1	21381
**31mar2020**	71	43	77	18	12	29	38	187714	1	25839
**01apr2020**	68	42	73	18	11	28	37	213057	1	25343
**02apr2020**	68	39	74	20	11	25	37	243354	1	30297
**03apr2020**	61	38	70	19	11	25	38	275181	1	31827
**04apr2020**	62	39	76	19	12	30	36	308052	1	32871
**05apr2020**	60	36	76	20	12	33	39	335782	1	27730
**06apr2020**	58	37	72	19	12	30	36	365284	1	29502
**07apr2020**	58	34	64	18	13	28	38	395884	1	30600
**08apr2020**	55	31	66	16	13	31	36	427297	1	31413
**09apr2020**	51	30	62	18	11	28	33	461900	1	34603
**10apr2020**	48	29	62	15	12	32	33	495215	1	33315

**11apr2020**	47	30	68	20	13	35	36	525045	1	29830
**12apr2020**	47	25	73	19	12	35	32	553398	1	28353
**13apr2020**	43	24	64	18	14	31	34	578605	1	25207
**14apr2020**	44	23	60	18	14	31	33	605517	1	26912
**15apr2020**	41	22	54	15	12	29	30	634452	1	28935
**16apr2020**	41	23	56	17	13	30	32	665528	1	31076
**17apr2020**	38	22	51	17	11	29	30	698100	1	32572
**18apr2020**	39	21	61	16	11	33	36	726178	1	28078
**19apr2020**	39	22	61	18	13	33	36	752014	1	25836
**20apr2020**	39	20	56	16	12	33	34	779182	1	27168
**21apr2020**	38	20	57	17	11	32	34	804462	1	25280
**22apr2020**	39	19	54	16	11	31	31	832600	1	28138
**23apr2020**	38	19	56	16	12	31	32	866481	1	33881
**24apr2020**	36	19	51	16	12	31	29	902743	1	36262
**25apr2020**	39	20	57	17	12	33	34	935461	1	32718
**26apr2020**	34	18	59	18	11	35	34	962941	1	27480
**27apr2020**	35	18	56	17	12	31	33	985348	1	22407
**28apr2020**	35	18	53	15	13	30	33	1009760	1	24412
**29apr2020**	35	17	57	15	11	31	33	1037023	1	27263
**30apr2020**	33	18	53	15	13	32	32	1066428	1	29405
**01may2020**	31	16	52	14	13	28	30	1100219	1	33791
**02may2020**	31	17	56	15	11	34	32	1129093	1	28874
**03may2020**	34	16	62	18	10	36	37	1154410	1	25317
**04may2020**	32	15	54	17	11	31	31	1176696	1	22286
**05may2020**	30	16	58	16	11	31	31	1200648	1	23952
**06may2020**	29	16	55	15	13	30	32	1225716	1	25068
**07may2020**	31	17	53	15	12	29	31	1253342	1	27626
**08may2020**	30	15	55	14	11	30	30	1280125	1	26783
**09may2020**	29	16	56	15	10	33	32	1305644	1	25519
**10may2020**	30	16	54	16	12	36	32	1325226	1	19582
**11may2020**	33	15	57	18	13	34	36	1343926	1	18700
**12may2020**	30	16	54	18	12	30	32	1365738	1	21812
**13may2020**	32	15	54	15	14	33	32	1386804	1	21066
**14may2020**	31	15	52	15	12	33	34	1414171	1	27367
**15may2020**	32	13	52	15	11	28	28	1439219	1	25048
**16may2020**	31	15	57	16	13	33	34	1464100	1	24881
**17may2020**	30	15	62	16	13	36	33	1482966	1	18866
**18may2020**	30	13	58	16	13	31	33	1504463	1	21497
**19may2020**	31	14	56	16	12	34	35	1524702	1	20239

**20may2020**	30	14	55	15	13	30	33	1548136	1	23434
**21may2020**	29	16	55	14	12	29	32	1573377	1	25241
**22may2020**	29	14	55	15	12	31	32	1597292	1	23915
**23may2020**	28	15	56	17	11	34	35	1618976	1	21684
**24may2020**	29	13	62	15	12	37	36	1639531	1	20555
**25may2020**	29	13	60	18	13	33	37	1658315	1	18784
**26may2020**	28	14	59	15	14	31	30	1676989	1	18674
**27may2020**	29	13	56	18	13	32	33	1695207	1	18218
**28may2020**	29	13	59	15	12	30	34	1717867	1	22660
**29may2020**	28	13	56	15	12	30	30	1742147	1	24280
**30may2020**	26	12	59	15	10	31	34	1766143	1	23996
**31may2020**	26	13	60	16	13	35	31	1786130	1	19987
**01jun2020**	26	12	56	16	12	29	31	1803327	1	17197
**02jun2020**	26	11	58	13	12	28	32	1824126	1	20799
**03jun2020**	27	11	51	15	11	28	31	1843786	1	19660
**04jun2020**	26	11	55	15	12	31	30	1864487	1	20701
**05jun2020**	26	12	56	14	12	32	33	1889607	1	25120
**06jun2020**	28	12	63	16	12	32	31	1911926	1	22319
**07jun2020**	29	14	64	15	12	35	34	1929563	1	17637
**08jun2020**	29	12	58	17	12	30	31	1946916	1	17353
**09jun2020**	28	12	60	17	12	32	36	1964776	1	17860
**10jun2020**	31	11	62	14	14	32	31	1985402	1	20626
**11jun2020**	27	12	59	17	13	32	33	2008212	1	22810
**12jun2020**	28	12	58	16	11	30	32	2033359	1	25147
**13jun2020**	28	13	61	16	11	31	35	2058690	1	25331
**14jun2020**	27	13	66	19	12	35	36	2078302	1	19612
**15jun2020**	29	13	62	17	13	32	37	2097898	1	19596
**16jun2020**								2121522	1	23624
**17jun2020**								2146997	1	25475
**18jun2020**								2174591	1	27594
**19jun2020**								2205977	1	31386
**20jun2020**								2238373	1	32396
**21jun2020**								2264630	1	26257
**22jun2020**								2295200	1	30570
**23jun2020**								2330229	1	35029
**24jun2020**								2364895	1	34666
**25jun2020**								2404770	1	39875
**26jun2020**								2449937	1	45167
**27jun2020**								2492359	1	42422

**28jun2020**								2531049	1	38690
**29jun2020**								2572494	1	41445
