# Supplementary figures and images for: Association of Search Query Interest in Gastrointestinal Symptoms With COVID-19 Diagnosis in the United States: Infodemiology Study
Source: JMIR Public Health Surveill. 2020 Jul 17;6(3):e19354. doi: 10.2196/19354 (PMC7371406; doi:10.2196/19354)

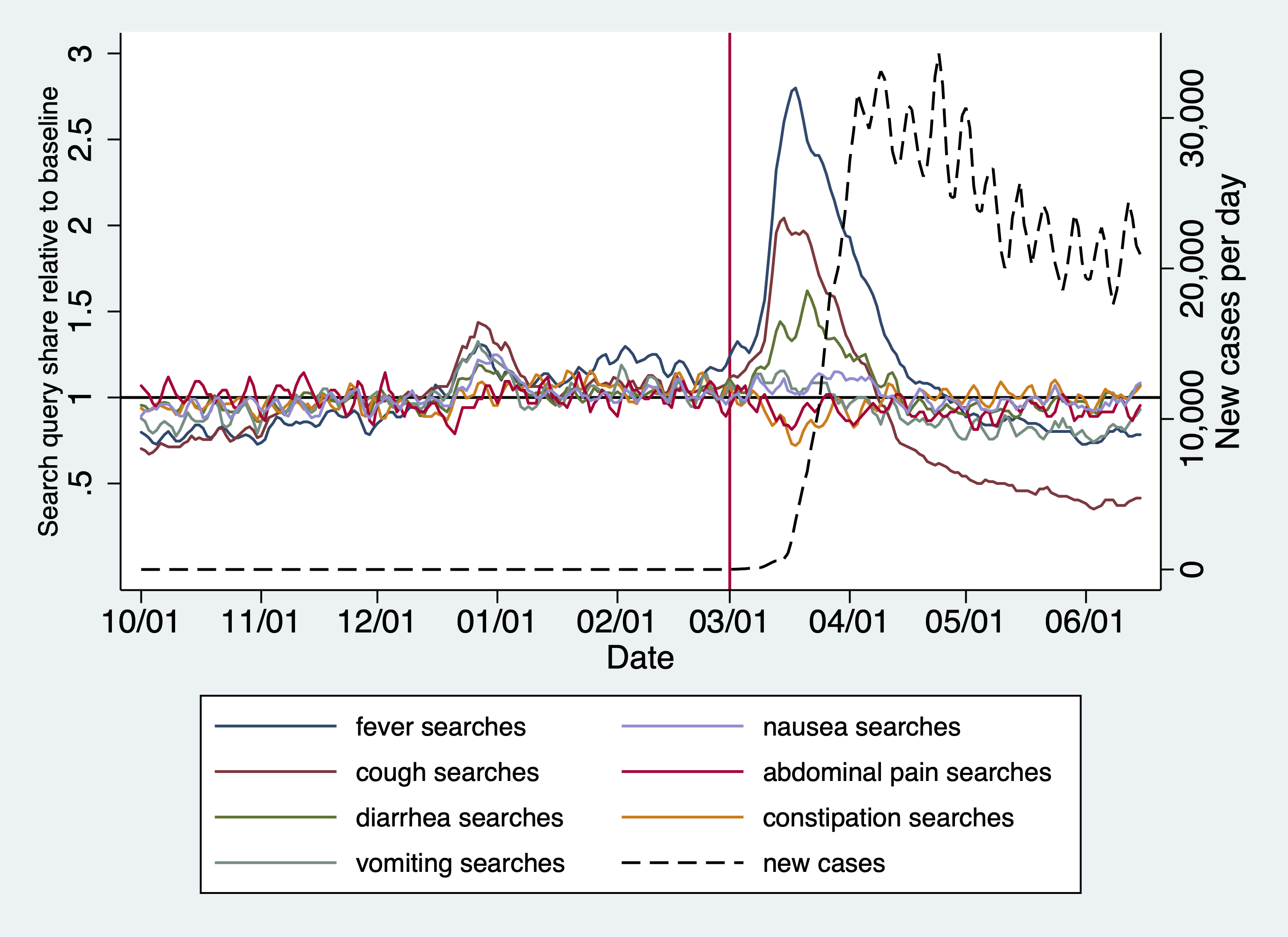

Supplement: Multimedia Appendix 2 [file publichealth_v6i3e19354_app2.png]
